# Supplementary material for: Preparation of 3D Nd2O3-NiSe-Modified Nitrogen-Doped Carbon and Its Electrocatalytic Oxidation of Methanol and Urea
Source: Nanomaterials (Basel). 2023 Feb 22;13(5):814. doi: 10.3390/nano13050814 (PMC10005539; doi:10.3390/nano13050814)
Supplement: Supplementary file 1 [file nanomaterials-13-00814-s001.zip › nanomaterials-2200371-supplementary.pdf]

nanomaterials

MDPI

Supporting Materials

Preparation of 3D Nd_2O_3-NiSe Modified Nitrogen Doped Carbon and Its Electrocatalytic Oxidation of Methanol and Urea

Simin Zhang, Ying Chang, Aiju Xu, Jingchun Jia * and Meilin Jia *

Inner Mongolia Key Laboratory of Green Catalysis and Inner Mongolia Collaborative Innovation Center for Wa-ter Environment Safety, College of Chemistry and Environmental Science, Inner Mongolia Normal University, Hohhot 010022, China

* Correspondence: jjc1983@126.com (J.J.); jml@imnu.edu.cn (M.J.)

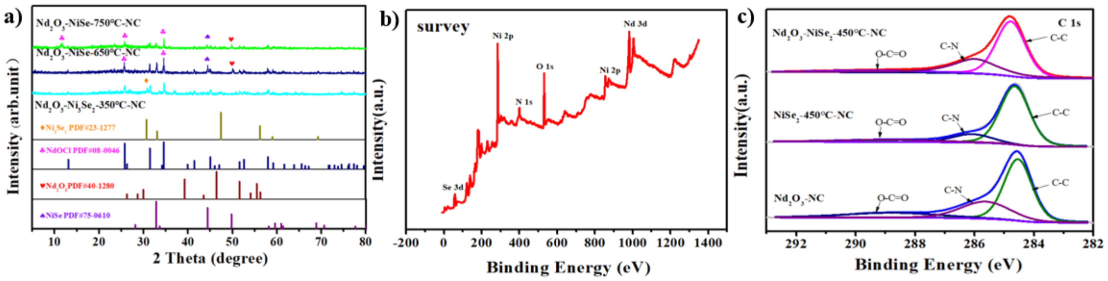

Figure S1. (a) XRD patterns of Nd_2O_3-Ni_3Se_2-350°C-NC, Nd_2O_3-NiSe-650°C-NC and Nd_2O_3-NiSe-750°C-NC; XPS spectrum tests of (b) Survey; (c) C 1s.

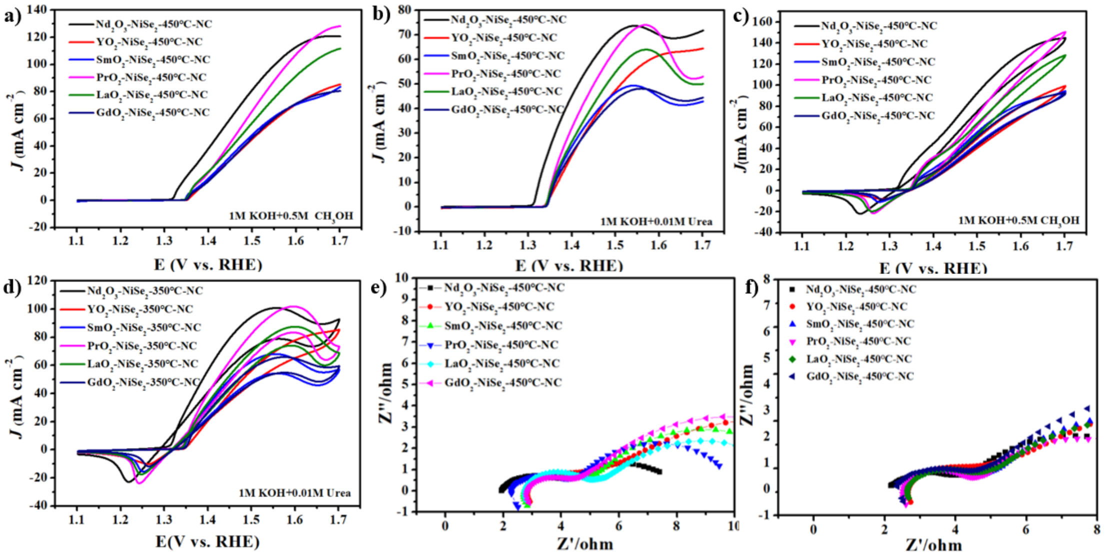

Figure S2. (a) LSV of Nd_2O_3-$NiSe_2$-450°C-NC, YO_2-$NiSe_2$-450°C-NC, SmO_2-$NiSe_2$-450°C-NC, PrO_2-$NiSe_2$-450°C-NC, LaO_2-$NiSe_2$-450°C-NC and GdO_2-$NiSe_2$-450°C-NC in 1 M KOH containing 0.5 M MeOH ; (b) LSV of Nd_2O_3-$NiSe_2$-450°C-NC, YO_2-$NiSe_2$-450°C-NC, SmO_2-$NiSe_2$-450°C-NC, PrO_2-$NiSe_2$-450°C-NC, LaO_2-$NiSe_2$-450°C-NC and GdO_2-$NiSe_2$-450°C-NC in 1 M KOH containing 0.01 M Urea; (c) CVs of Nd_2O_3-$NiSe_2$-450°C-NC, YO_2-$NiSe_2$-450°C-NC, SmO_2-$NiSe_2$-450°C-NC, PrO_2-$NiSe_2$-450°C-NC, LaO_2-$NiSe_2$-450°C-NC and GdO_2-$NiSe_2$-450°C-NC in 1 M KOH containing 0.5 M MeOH (sweep speed: 50 mV s^{-1}); (d) CVs of Nd_2O_3-$NiSe_2$-450°C-NC, YO_2-$NiSe_2$-450°C-NC, SmO_2-$NiSe_2$-450°C-NC, PrO_2-$NiSe_2$-450°C-NC, LaO_2-$NiSe_2$-450°C-NC and GdO_2-$NiSe_2$-450°C-NC in 1 M KOH containing 0.01 M Urea (sweep speed: 50 mV s^{-1}); (e) EIS of Nd_2O_3-$NiSe_2$-450°C-NC, YO_2-$NiSe_2$-450°C-NC, SmO_2-$NiSe_2$-450°C-NC, PrO_2-$NiSe_2$-450°C-NC, LaO_2-$NiSe_2$-450°C-NC and GdO_2-$NiSe_2$-450°C-NC in 1 M KOH containing 0.5 M MeOH; (f) EIS of Nd_2O_3-$NiSe_2$-450°C-NC, YO_2-$NiSe_2$-450°C-

NC, SmO_2-$NiSe_2$-450°C-NC, PrO_2-$NiSe_2$-450°C-NC, LaO_2-$NiSe_2$-450°C-NC and GdO_2-$NiSe_2$-450°C-NC in 1M KOH containing 0.01 M Urea.

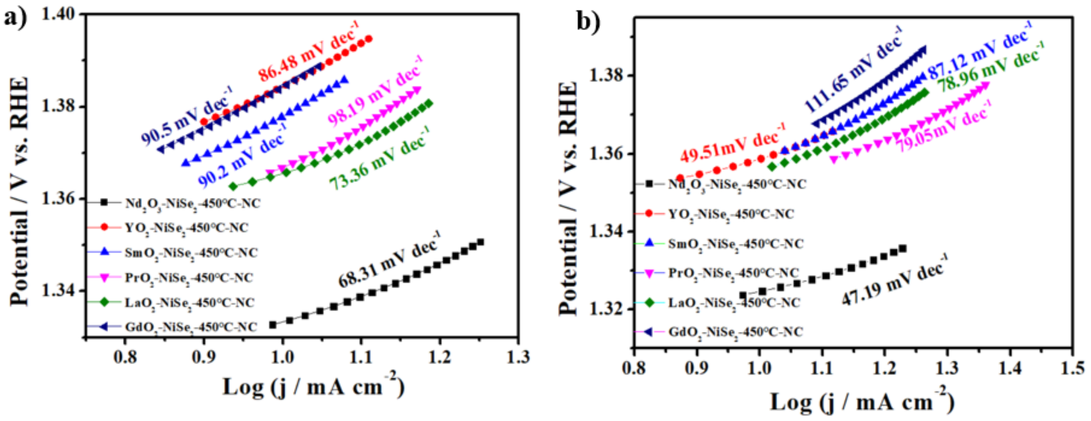

Figure S3. The Tafel plots of different samples in methanol (a) and urea (b) oxidation.

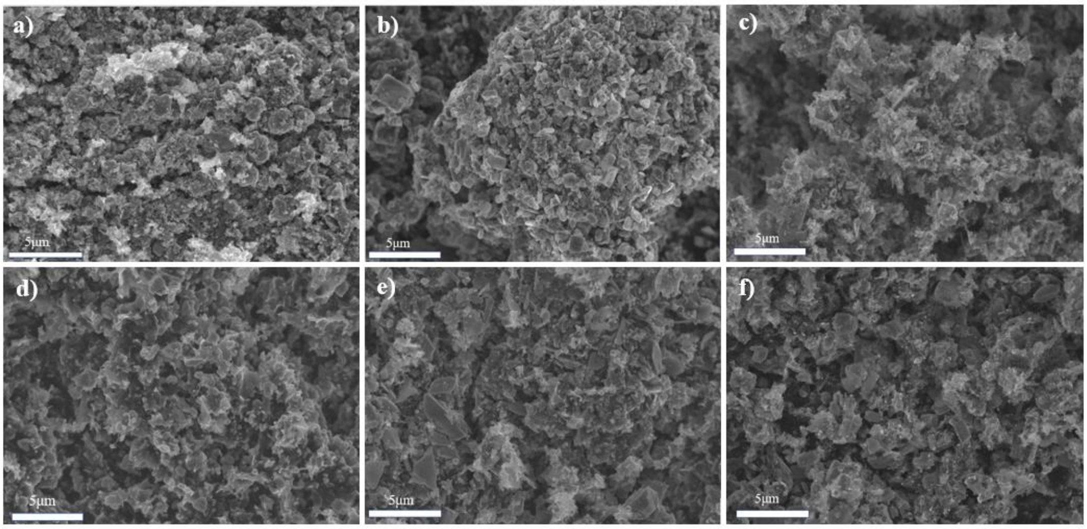

Figure S4. (a) SEM images of YO_2-$NiSe_2$-450°C-NC, (b) SEM images of LaO_2-$NiSe_2$-450°C-NC, (c) SEM images of GdO_2-$NiSe_2$-450°C-NC, (d) SEM images of PrO_2-$NiSe_2$-450°C-NC, (e) SEM images of SmO_2-$NiSe_2$-450°C-NC, (f) SEM images of Nd_2O_3-$NiSe_2$-450°C-NC.

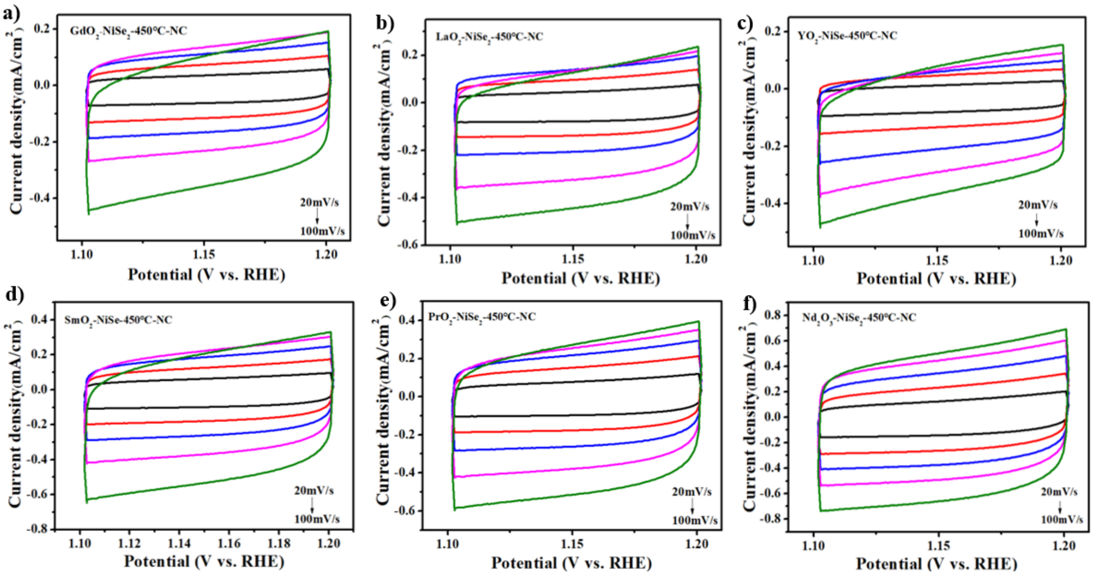

Figure S5. (a-f) GdO2-NiSe2-450°C-NC, LaO2-NiSe2-450°C-NC, YO2-NiSe2-450°C-NC, SmO2-NiSe2-450°C-NC, PrO2-NiSe2-450°C-NC, Nd2O3-NiSe2-450°C-NC at scan rate of 20, 40, 60, 80 and 100 mV s-1 in 1 M KOH solution.

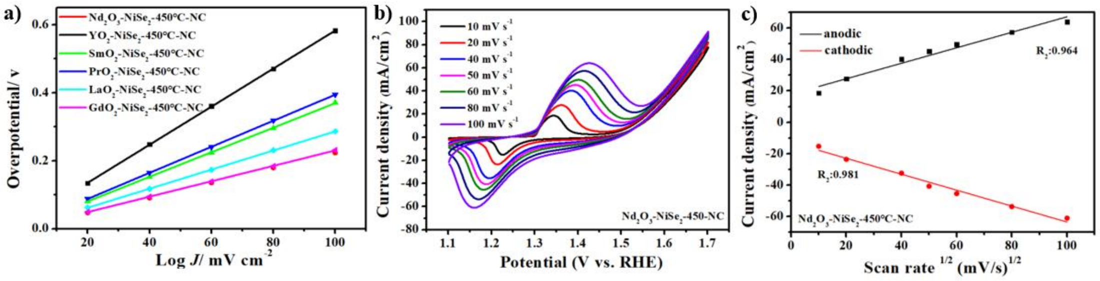

Figure S6. (a) the Cdl of nickel based catalysts with different rare earth metals; (b) CV of different sweep speeds (10–100 mV s−1) for Nd2O3-NiSe2-450°C-NC; (c) linear fitting of the anodic and cathodic peak current densities with the square roots of the scan rate in the scan rate range (10–100 mV s−1) for Nd2O3-NiSe2-450°C-NC.

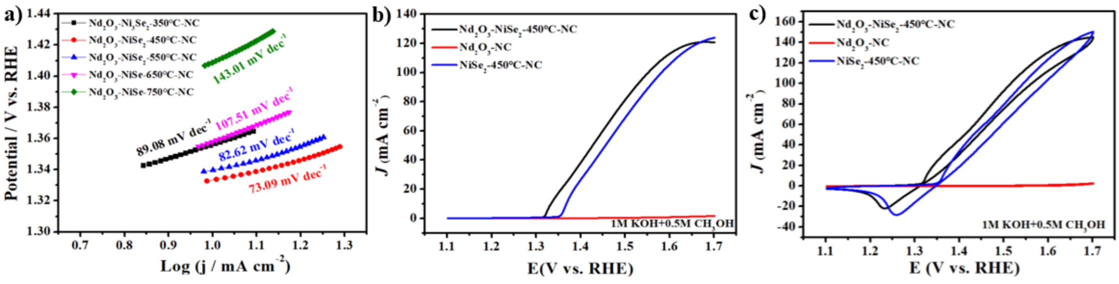

Figure S7. (a) The Tafel plots of different samples in methanol; (b) LSV of Nd2O3-NiSe2-450°C-NC, Nd2O3-NC and NiSe2-450°C-NC in 1 M KOH containing 0.5 M MeOH. (c) CVs of Nd2O3-NiSe2-450°C-NC, Nd2O3-NC and NiSe2-450°C-NC in 1M KOH containing 0.5 M MeOH (sweep speed: 50 mV s−1).

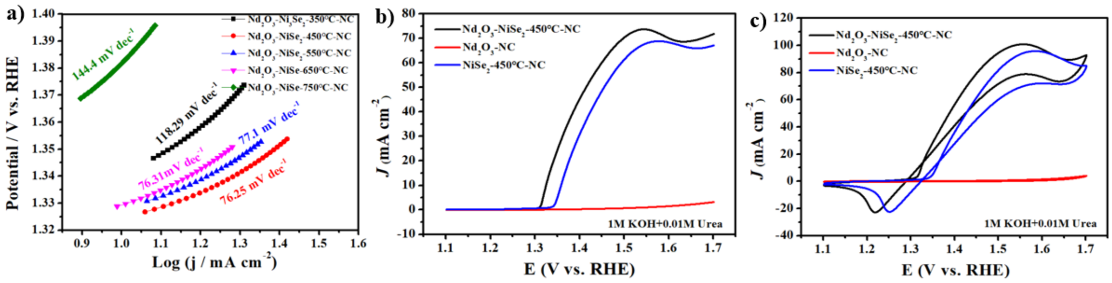

Figure S8. (a) The Tafel plots of different samples in Urea; (b) LSV of Nd_2O_3-$NiSe_2$-450°C-NC, Nd_2O_3-NC and $NiSe_2$-450°C-NC in 1M KOH containing 0.01 M Urea (sweep speed: 50 mV s^{-1}); (c) CVs of Nd_2O_3-$NiSe_2$-450°C-NC, Nd_2O_3-NC and $NiSe_2$-450°C-NC in 1 M KOH containing 0.01 M Urea (sweep speed: 50 mV s^{-1}).
